# Comparison Between the Yield of Different Number of Blood Cultures in Chronic Kidney Disease Patients With Suspected Septicemia

**DOI:** 10.7759/cureus.20381

**Published:** 2021-12-13

**Authors:** Faiza Anser, Murtaza Dhrolia, Safia Qureshi, Kiran Nasir, Ruqaya Qureshi, Aasim Ahmad

**Affiliations:** 1 Nephrology, The Kidney Centre Postgraduate Training Institute, Karachi, PAK; 2 Microbiology, The Kidney Centre Postgraduate Training Institue, Karachi, PAK; 3 Nephrology, The Kidney Centre Postgraduate Teaching Institute, Karachi, PAK

**Keywords:** infections, sepsis, septicemia, chronic kidney disease, blood culture

## Abstract

Objective: Our study aimed to evaluate the optimal and financially efficient numbers of blood cultures (BC) required in our chronic kidney disease (CKD) patients with suspected bloodstream infections (BSI).

Design: This is a prospective, cross-sectional study.

Place and duration of study: Department of Nephrology, The Kidney Center Post-Graduate-Training-Institute, Karachi from July 2020 to December 2020.

Methods: Single, two, or three BC were taken from CKD patients with suspected BSI within the first 24 hours and were incubated in the BACTEC 1050 CMBCS for five days. A positive culture was reported as per standard protocol.

Results: Four hundred and eighty-three BC sets were collected from 272 patients. A single set of BC was obtained from 111 (40.8%), two sets from 111 (40.8%), and three from 50 (18.4%) patients. BC from 93 patients showed growth of organisms in at least one set. Fifty-six (60.2%) episodes of BSI were detected with the first set, 34 (36.5%) with the second set, and 03 (3.2%) with the third set of BC. The detection rate of BSI was 60.2% with the first set, 97.7% with the first two sets, and 100% with the first three sets of BC. The most common source of infection was central line-associated bloodstream infection (CLABSI) (33.3%), followed by urinary tract (29%), lower respiratory tract infection (LRTI) (16%), and arteriovenous fistula (AVF) (7.5%). 93.5% episodes of BSI, were monomicrobial. The most common monomicrobial organism was methicillin-resistant *Staphylococcus aureus *(MRSA) (22.6%).

Conclusion: Two properly collected BC sets might be sufficient for an adequate diagnosis of BSI, in CKD patients especially in resource-limited settings.

## Introduction

Infection is the most preventable and common cause of non-cardiovascular morbidity and mortality among the pre-dialysis chronic kidney disease (CKD) patients [1,2] and the second leading cause of death [3], and substantial morbidity [4], in patients on HD. The leading risk factor of bacteremia in chronic HD patients is vascular access especially central venous catheters [5]; rates of hospitalization secondary to BSI in patients with pre-dialysis CKD were also threefold to fourfold greater than in patients without CKD [6]. Complications related to bloodstream infection (BSI) in patients with CKD are also a significant source of morbidity and mortality [1]; therefore, it is important to have an accurate and prompt diagnosis of BSI in CKD patients.

The etiology of the increased risk of BSI in CKD patients may be multifactorial. Renal dysfunction may itself be caused by other conditions that increase vulnerability to infection such as age, diabetes, other co-morbidities, malignant neoplasms, urologic disease, or immunosuppressant use. Alternatively, malnutrition, chronic inflammation, retention of uremic solutes, trace element deficiencies, and metabolic abnormalities, associated with CKD may predispose these patients to infection [7] along with impairment of immune function [8].

Timely identification of bloodstream infections and accurate antimicrobial therapy play a major role in the cure of these infections. Blood culture (BC) is the gold standard test to diagnose infections [9]. Weinstein et al. [10] in 1983 reported detection of 99.3% episodes with the first two BC. It is thereby recommended by most guidelines that at least two or three sets of BC should be collected [11,12]. Further, there have been numerous changes in BC media and systems over time. Elantamilan et al. [13] gave a sensitivity of one BC as 85.67%, the first two BCs as 96.59%, and 100% for the first three BCs using BacT/ALERT 3D-automated blood culture system (BCS).

However, regardless of the recommended guidelines, the actual practice of obtaining blood culture differs significantly, either in the quantity of blood sampled per culture or in the number of samples obtained for culture especially in resource-limited countries like Pakistan (mainly due to financial constraints) [14]. In contrast to developed countries, where the emphasis is on improving the quality of life and long-term survival through effective health care, the enormous cost of therapy limits the continuation of treatment in our region [15] and it is common for HD patients to compromise the prescribed therapy or investigations as financial resources decline, usually leading to discontinuation of dialysis and death. Considering this, along with the risk of delaying antimicrobial therapy and high-risk mortality, in HD patients with suspected sepsis or septic shock, the practice of collection of blood cultures is still not according to the recommended guidelines especially in the HD patients in this part of the world.

There are no previously published reports from Pakistan that estimate the yield of single versus multiple blood cultures for the detection of pathogenic organisms in CKD patients with septicemia. Our study aimed to compare the yield of different numbers of blood cultures in CKD patients with suspected septicemia to evaluate the optimal and financially efficient numbers of blood cultures required in our CKD patients to detect BSIs. Accurate and prompt detection of BSIs may benefit patient care by prescribing a rational antibiotic, reducing the length of hospitalization, and eventually increasing patient survival.

## Materials and methods

This observational, cross-sectional study was conducted in the Department of Nephrology, The Kidney Centre Postgraduate Training Institute (TKC-PGTI) after approval by the institutional ethical review committee. Patients with CKD of both gender, aged >18 years, admitted in hospital for more than 24 hours, registered either via emergency or outpatient department, with two or more of the following clinical features suggestive of BSIs; temperature >38 °C or <36 °C, heart rate >90/min, respiratory rate >20/min or PaCO_2_ <32 mm Hg, white blood cell count >12,000/mm^3^ or <4000/mm^3^ or >10% immature bands according to systemic inflammatory response syndrome (SIRS) criteria 18 or with the presence of two or more of quick sepsis-related organ failure assessment (qSOFA) clinical criteria 19 (altered mentation, respiratory rate ≥22 breaths/min, and systolic blood pressure ≤100 mm Hg) requiring blood cultures were enrolled in the study through non-probability consecutive sampling. Informed written consent was taken. Patients already on antimicrobial therapy, and those with malignancy were excluded.

The numbers of blood cultures sent within the first 24 hours of the patient’s hospital admission were at the discretion of the attending doctor and treating physician. The primary outcome measure was to compare the yield of a different number of blood cultures in CKD patients with suspected septicemia. Blood specimens were obtained by nursing staff or by trained phlebotomists observing all aseptic measures for blood sample collection at the same time before the start of antimicrobial therapy with or without fever spikes preferably from peripheral venipuncture. For patients with double lumen catheter in which two or more sets of BC were taken, one sample was collected from the central line and the other one or two from the peripheral vein. For each set of blood cultures, 20-30 ml of blood was taken and inoculated into aerobic BACTEC bottles. Due to financial constraints, anaerobic cultures were not done, as the clinical history of our patients did not suggest anaerobic infection. BC bottles were analyzed according to the standard routine of our microbiology department using BACTEC 1050 CMBCS (as per standard guidelines recommended by the manufacturer). BC bottles were incubated in the BACTEC for a maximum of five days. When the bottle was flagged positive by the BACTEC, microscopy followed by culture and susceptibility testing was performed. Organisms were identified using conventional microscopic examination by Gram’s stain, and routine culture media and biochemical reactions. A positive culture was reported as per the standard routine of our hospital.

Statistical analysis

Data were entered and analyzed by IBM version 21 of SPSS (IBM Corp., Armonk, NY). Mean with standard deviation and median with interquartile range (IQR) were calculated for continuous variables, while for categorical variables, frequencies with percentage were obtained.

## Results

Four hundred and eighty-three BC sets were collected from 272 patients. The mean age of our patients was 51.1 ± 16.4 years; 168 (61.8%) were male and 104 (38.2%) were female. Most common comorbidity was hypertension (n=250 [91.9%]) followed by diabetes mellitus (n=70 [25.7%]), ischemic heart disease (n=20(7.4%]), and congestive cardiac failure (n= 10 [3.4%]). Seventy-nine (29%) of our study participants were of CKD stage II-III, 42 (15.4%) CKD stage IV, and 151 (55.5%) CKD stage V; 66 (24.3%) of our patients were on HD. Demographic and clinical characteristics of study participants are presented in Table 1.

**Table 1:** Demographic and clinical characteristics of participants CKD: chronic kidney disease, ADPKD: autosomal dominant polycystic kidney disease

Variables		n(%)/mean ± std
Gender	Male	168(61.8)
	Female	104(38.2)
Age		51.1 ± 16.4
Comorbid	Hypertension	250(91.9)
	Diabetes mellitus	70(25.7)
	Ischemic heart disease	20(7.4)
	Congestive cardiac failure	10(3.4)
Stage of CKD	II-III	79(29)
	IV	42(15.4)
	V	151(55.5)
On hemodialysis		66(24.3)
Cause of CKD	Unknown	125(46)
	Diabetes mellitus	70(25.7)
	Renal stone	33(11.4)
	Glomerulonephritis	31(11.4)
	ADPKD	13(4.8)

Among the 483 BC sets from 272 patients, a single set of BC was obtained from 111 (40.8%) patients, two sets from 111 (40.8%) patients, and three from 50 (18.4%) patients. BC from 93 patients showed growth of organisms in at least one set, defining episode of BSI. Seventeen (15.3%) out of 111, 59 (53.2%) of 111, and 17 (34%) of 50 patients grew organisms in which a single, two, or three sets of BC was obtained. Fifty-six (60.2%) episodes of BSI were detected with the first set, 34 (36.5%) with the second set, and 03 (3.2%) with the third set of BC. The detection rate of BSI was found to be 60.2% with the first set of BC, 97.7% with the first two sets of BC, and 100% with the first three sets of BC (Table 2).

**Table 2:** Detection rate of bloodstream infection from different numbers of blood cultures BSI: bloodstream infection, BC: blood culture

	Patient n (%)	Episodes of BSI detected n (%)		Detection rate of BSI %	
Total BC	272 (100)	Total	93 (34.2)		
One set of BC	111 (40.8)	With the first set	56 (60.2)	With the first set	60.2
Two sets of BC	111 (40.8)	With the second set	34 (36.5)	With the first 2 sets	97.7
Three sets of BC	50 (18.4)	With the third set	3 (3.2)	With the first 3 sets	100

The most common source of infection in patients with positive culture was central line-associated bloodstream infection (CLABSI) 31 (33.3%), followed by urinary tract infection (UTI) 27 (29%), lower respiratory tract infection (LRTI) 15 (16%), and arteriovenous fistula (AVF) 7 (7.5%) (Table 3). Seventeen out of 79 patients with CKD stage II-III, 15 of 42 CKD stage IV, and 61 of 151 CKD stage V had positive BC. Forty-one out of 66 patients on HD grew organisms. The most common source of infection in patients on HD was CLABSI (75.6%) followed by AVF (17%) and LRTI (7.3%).

**Table 3:** Suspected source of infection LRTI: lower respiratory tract infection, UTI: urinary tract infection, AVF: arteriovenous fistula, CLABSI: central line-associated bloodstream infection

BC result	unknown	LRTI	UTI	AVF	CLABSI	Total
Negative	52	46	62	2	17	179
Positive	13	15	27	7	31	93
Total	65	61	89	9	48	272

Out of 93 episodes of BSI, 87 (93.5%) were monomicrobial and 06 (6.5%) were polymicrobial. Microorganisms isolated from these BSI episodes are shown in Table 4. The most common monomicrobial organism was methicillin-resistant *Staphylococcus aureus* (MRSA) (n= 21 [22.6%]), followed by *Escherichia coli* (n=17 [18.3%]). *Candida albicans* was recovered from (n=3 [3.2%]) blood cultures.

**Table 4:** Microorganisms isolated from monomicrobial and polymicrobial BSIs n(%) BSI: bloodstream infection

Total positive cultures	93(34.2)
Monomicrobial BSIs	87
Gram-positive	
Methicillin-resistant *S. aureus*	21(22.6)
Staphylococcus spp.	14(15)
Methicillin-sensitive *S. aureus*	7(7.5)
Corynebacterium spp	4(4.3)
Enterococcus spp.	2(2.1)
Gram-negative	
*E. coli*	17(18.3)
*Psuedomonas aeruginosa*	13(14)
Acinetobacter spp.	3(3.2)
*Klebsiella pneumoniae*	2(2.1)
*Salmonella typhi*	1(1)
*Candida albicans*	3(3.2)
Polymicrobial BSIs	6
*E. coli*, Staphylococcus spp.	1
​​​​​​​*E. coli*, Enterococcus spp.	1
​​​​​​​*P. aeruginosa*, Staphylococcus spp.	1
​​​​​​​*E. coli*, *K. pneumoniae*	1
​​​​​​​*E. coli*, *P. aeruginosa*	1
​​​​​​​*K. pneumoniae*, Acinetobacter spp.	1

## Discussion

Increased vulnerability to infection, impaired immune function [8], along significant morbidity and mortality associated with BSI in CKD patients [1] emphasize timely diagnosis and prompt treatment of BSI in this group of patients. BC remains the gold standard to diagnose BSI with various studies that have been done to evaluate the correct numbers [10], volume [9], time interval [9,14], and site [9] for sampling blood to determine the most efficient and accurate way for the diagnosis of BSI; however, to best of our knowledge, none have been done on patients with CKD.

In this study, we compared the yield of different numbers of blood cultures in CKD patients with suspected septicemia to evaluate the optimal and financially efficient numbers of blood cultures required in our CKD patients to detect BSIs. We evaluated 483 blood cultures received from 272 patients. A single set of blood cultures was received from 111 (40.8%) patients yielding a detection rate of 60.2%. While two sets of blood cultures were received from 111 (40.8%) patients. This increased the detection rate to 97.7%. Three sets of blood cultures, received from 50 (18.4%) patients, increased the detection rate to 100%. Thus, our findings remain consistent with the historical findings of Washington [10] and Weinstein et al. [10] both of whom found an increased yield with the increasing numbers of blood cultures. Elantamilan et al. [13] in their study from India, whose climatic and demographic factors are similar to ours, also found an increased detection rate of BSI with increasing numbers of BC sets in general hospital and intensive care unit patients.

In our study, we collected the different sets of blood cultures at the same time before the start of antimicrobial therapy with or without fever spikes mainly from peripheral venipuncture except for patients with double lumen catheter in which at least one sample was collected from the central line. Many studies support that volume is more important than timing or site of sampling in the detection of agents of septicemia [9,13].

In our study, 93 patients out of 272 showed growth of organisms in at least one set, making 34.2% correct suspicion of BSI in the study population which is better than the results from previous studies. Plausible explanation attributes to the correct clinical diagnosis of the treating physician in the suspicion of BSI in this group of patients, due to their increased vulnerability to infections and poor outcomes. Also, the resource-limited health setup in Pakistan forces the physician to make decisions for investigations that may be the least financially burdensome.

In our study, a single organism was detected in 87 (93.5%) blood cultures out of a total of 93 positive blood cultures reflecting true BSI. These included MRSA and methicillin-sensitive *S. aureus* (MSSA), Enterococcus spp. *S. typhi*, *E. coli*, *K. pneumoniae*, *P. aeruginosa*, Acinetobacter spp., and *C. albicans*. The most common Gram-positive pathogen in our study was MRSA 21 (43.7%) out of 48 gram-positive organism episodes with total episodes with Gram-positive organisms constituting 51.6% of the total episodes, i.e., 93. In a subgroup of patients on hemodialysis, 41 out of 66 patients grew organisms, of which 24 (58.5%) were Gram-positive organisms with 14 (34.1%) MRSA. This correlates with findings from other studies on hemodialysis patients that half to three-quarters of the causative organisms of bacteremia in hemodialysis patients are Gram-positive bacteria with MRSA, being the most common causative organism.

Corynebacterium spp. and Staphylococcus spp. being skin commensals might have reflected contamination at the time of sample collection when recovered from single blood culture in our patients. However, patients on hemodialysis or pre-dialysis CKD frequently have indwelling catheters and in these patients, these organisms, which are otherwise considered as cultural contaminants, might be significant pathogens, causing biofilm formation and leading to device-related bloodstream infections. These infections are characterized by their indolence, but they may necessitate the removal of the catheter and antibiotic therapy in these patients.

Overall, the most common source of infection in our study in patients with positive culture was double lumen catheter (DLC) reflecting CLABSI, followed by urinary tract, lower respiratory tract (LRT), and arteriovenous fistula (AVF). While the most common source of infection in the subgroup of patients on HD was DLC followed by AVF and LRT.

Our study was a single-center study with small sample size. Despite this, our study serves as a baseline data for future studies to be done in CKD patients especially in our part of the world (where data in general patients are also sparse) to evaluate the minimum number of blood cultures sufficient to correctly diagnose BSI in pre-dialysis and on-dialysis CKD patients, particularly in resource-limited health settings.

## Conclusions

Our data suggest that even two properly collected blood culture sets might be sufficient for an adequate diagnosis of BSI, in CKD patients especially in resource-limited health settings. The “single BC” practice, which is still common, is not acceptable, not only because of its unfavorable result when BSI is not detected and appropriately managed, due to wrong selection of antimicrobial treatment, but it also results in obliteration of the BSI source. We strongly recommend sending paired samples of blood cultures, from peripheral as well as an intravascular catheter for a definitive diagnosis of CLABSI in the absence of BSI suggestive of any other source.
